# Supplementary material for: Phylogeny and biogeography of the algal DMS-releasing enzyme in the global ocean
Source: ISME Commun. 2023 Jul 14;3:72. doi: 10.1038/s43705-023-00280-2 (PMC10349084; doi:10.1038/s43705-023-00280-2)
Supplement: Supplementary file 1 — Supplemental Material [file 43705_2023_280_MOESM1_ESM.pdf]

Supplementary Information

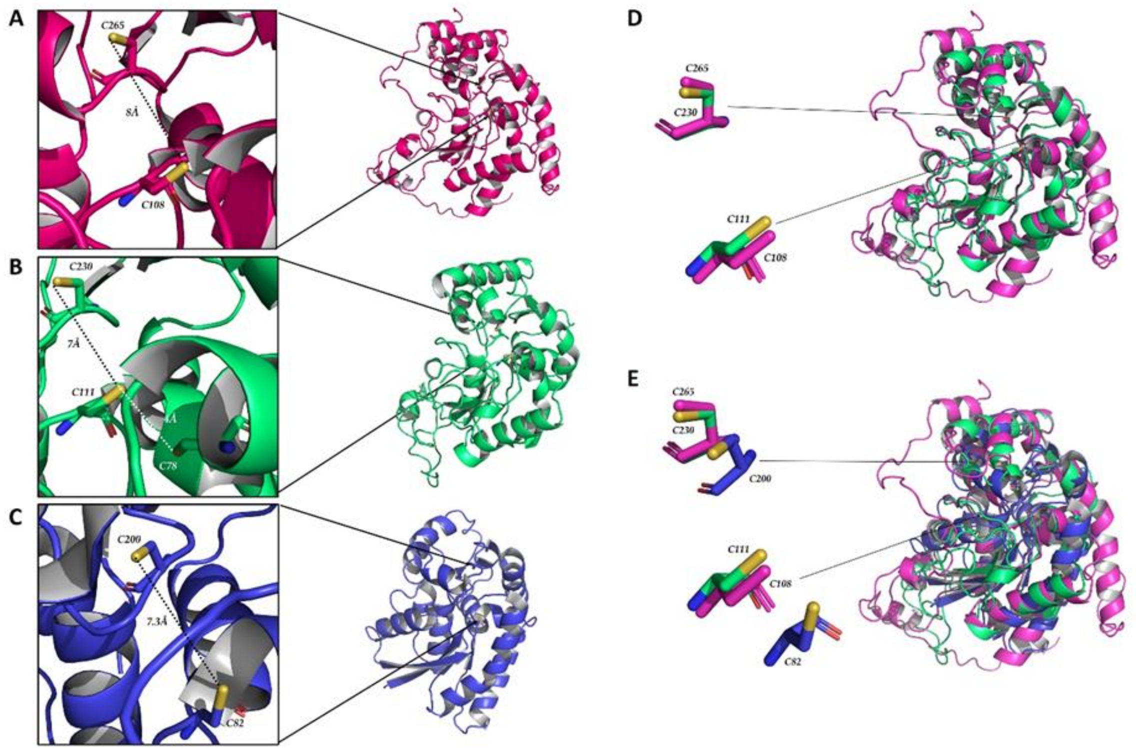

Fig. S1. Ribbon representation of the Alma1, Sym-Alma and bacterial Maleate Isomerase protein structures. (A-C) Predicted structure of two eukaryotic DMSP lyase proteins, Alma1 from *E. huxleyi* (A), and Sym-Alma from *Symbiodinium* A1 (B), as well as the known structure of bacterial Maleate Isomerase Iso (PDB:4FQ7) from *Pseudomonas putida* S16 (C). Insets show zoom-in view of the active site area in the DMSP lyases, where the canonical cysteines are shown as sticks: Cys108 and Cys265 for Alma1 (A) and Cys111 and Cys230 for Sym-Alma (B). (D-E) Structural alignment of Alma1 (pink) and Sym-Alma (green) (D) and Alma1, Sym-Alma and 4FQ7 (blue, E). Note that the cysteines residues of the eukaryotic homologs align perfectly, while the bacterial cysteines are not placed in the same position.

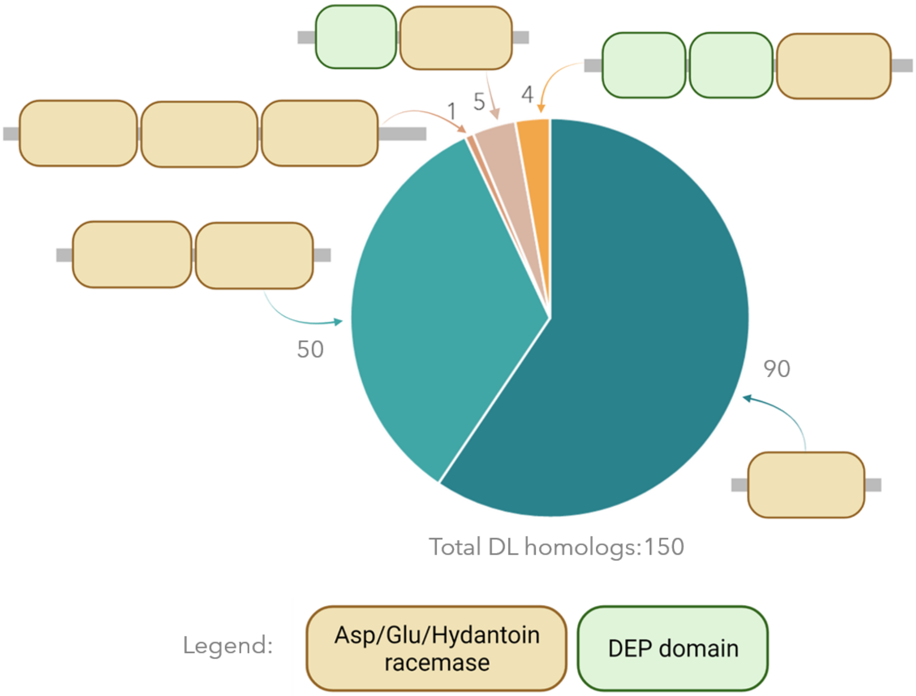

Fig. S2. Conserved domain organization of eukaryotic DL homologs. A schematic description of the different conserved domains (according to the CDD database) identified in a total of 150 DL homologs (see also dataset S1).

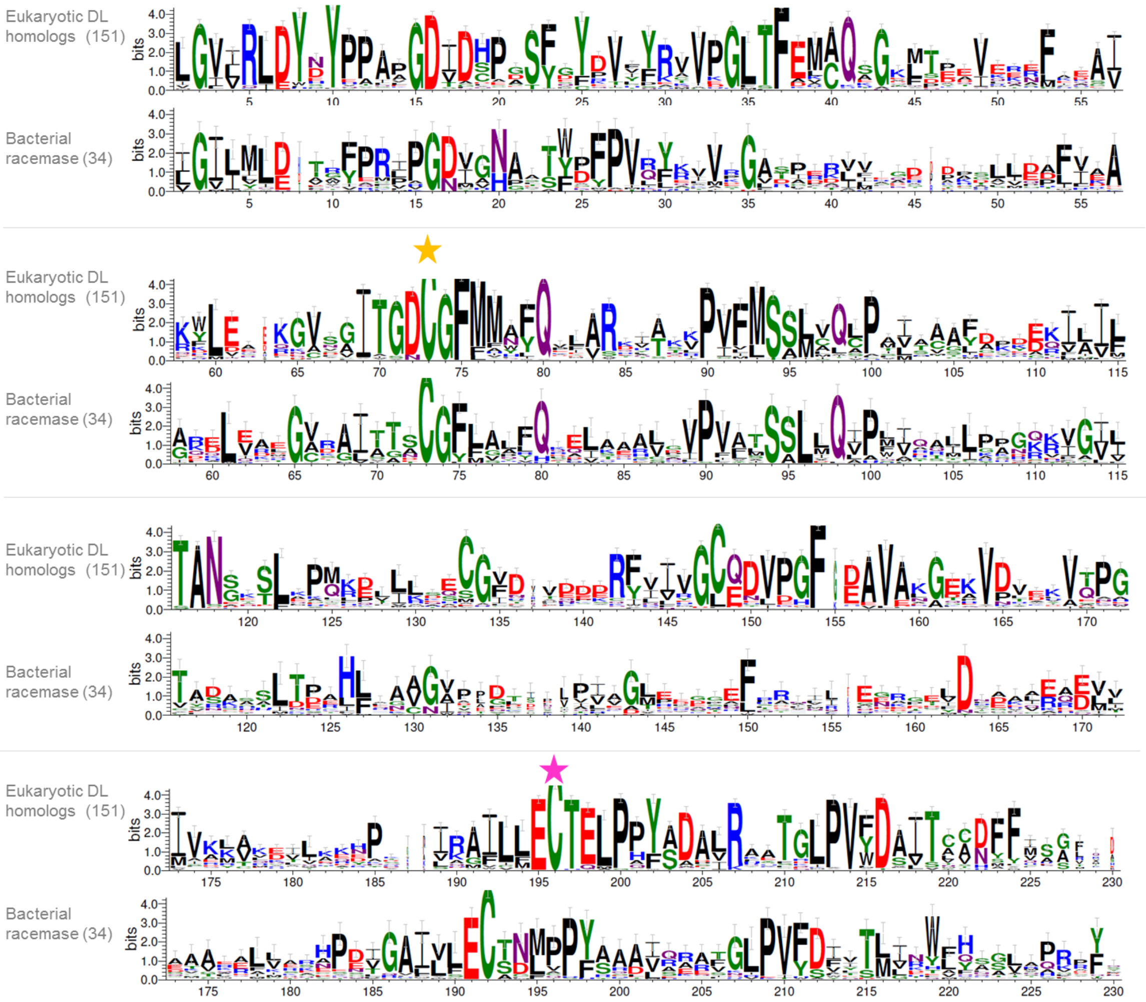

Fig. S3. Sequence logos of multiple sequence alignment of the racemase domain from bacteria and eukaryotic DL homologs. A total of 151 eukaryotic DL homologs were aligned (see dataset S1), and 34 bacterial sequences belong the CDD subfamily PRK0747 (which is the closest to the Asp/Glu/Hydantoin racemase domain). Stars designate the first (yellow) and second (pink) canonical cysteine residues located at the active site of the DL enzyme.

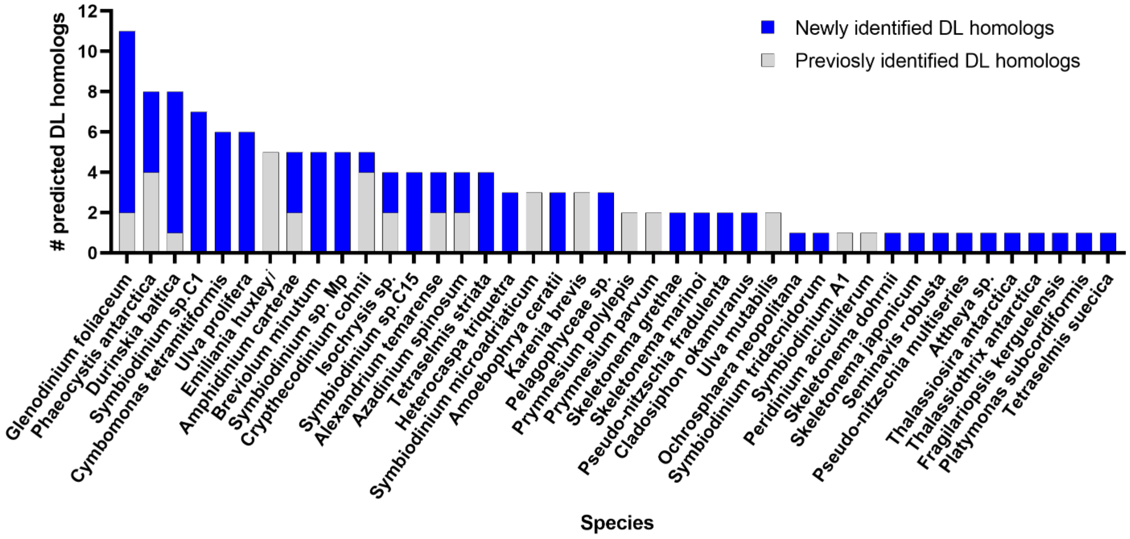

Fig. S4. Predicted DL homologs in different phytoplankton species. The number of predicted DL homologs is plotted per species.

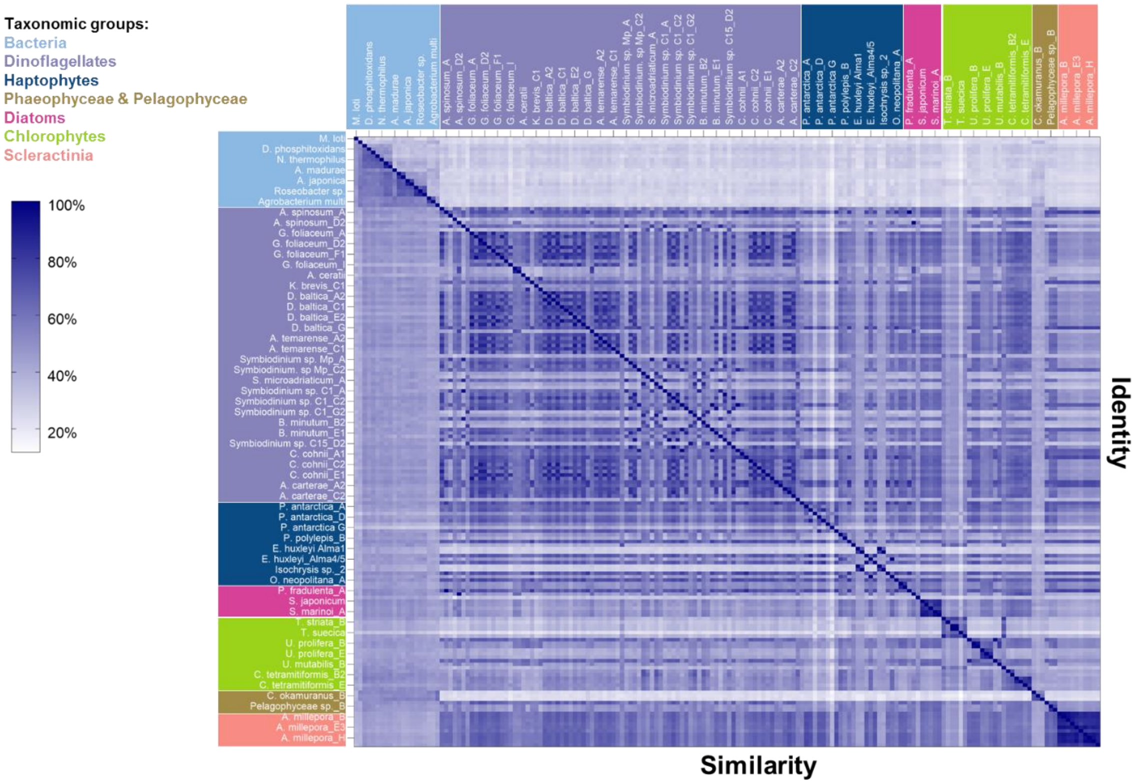

Fig. S5. Sequence similarity and identity of bacterial and eukaryotic DL homologs. The plot shows the sequence similarity scores below the diagonal and sequence identity scores above, for multiple sequence alignment of racemase domain of 174 DL homologs. Each row and column represent a DL homolog (see dataset S1). Due to space limitation, only third of the names could be presented. The sequences are orders according to their taxonomy (see legend).

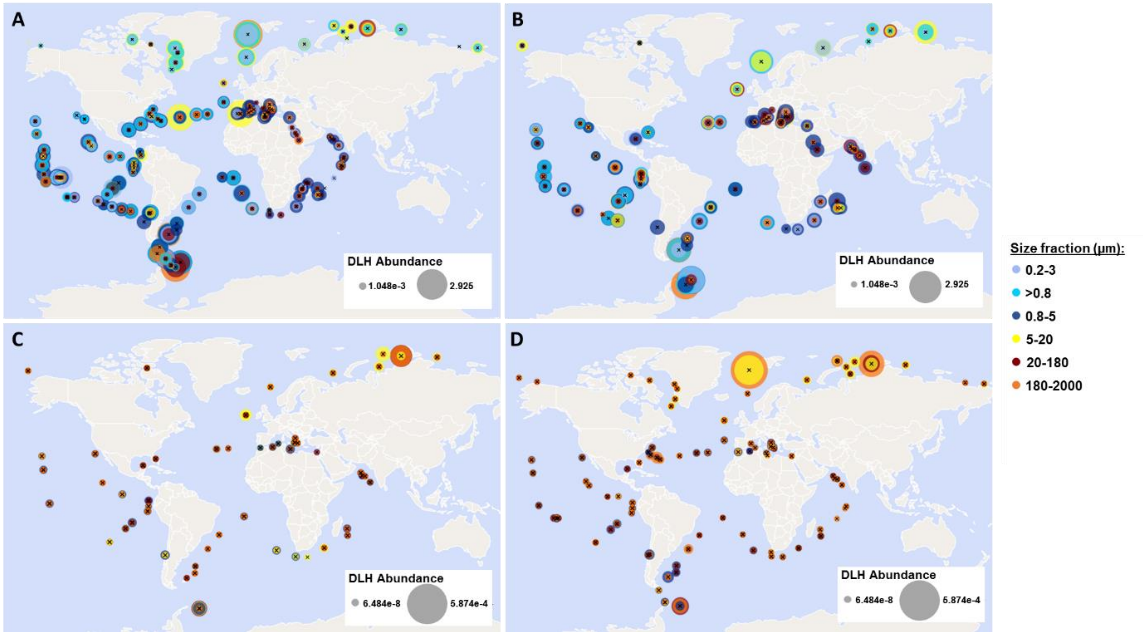

Fig. S6. Abundance of DMSP lyase homologs (DLHs) in the oceans. Geographic distribution of DLHs in phytoplankton found in *Tara* Oceans metagenomes (A,B) and metatranscriptomes (C,D) datasets. Color depicts the size fraction of each sample. The circle size is proportional to the relative abundance of DLH genes or transcripts, which was normalized as percent of mapped reads.

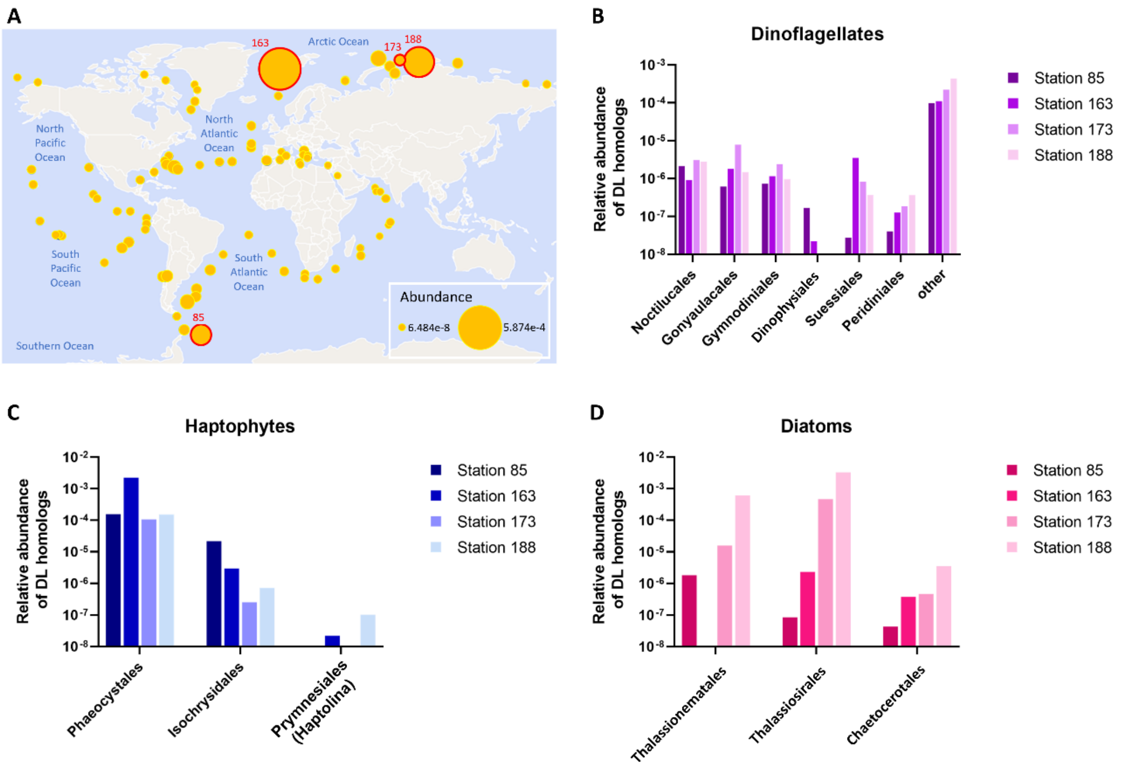

Fig. S7. Taxonomy of phytoplankton in selected *Tara* stations with high DL-homologs expression. (A). Location of stations *Tara* 85, 163, 173 and 188. The circle size is proportional to the relative abundance of DLH transcripts, which was normalized as percent of mapped reads. (B-D) Identified dinoflagellates, haptophytes and diatoms orders in each station.

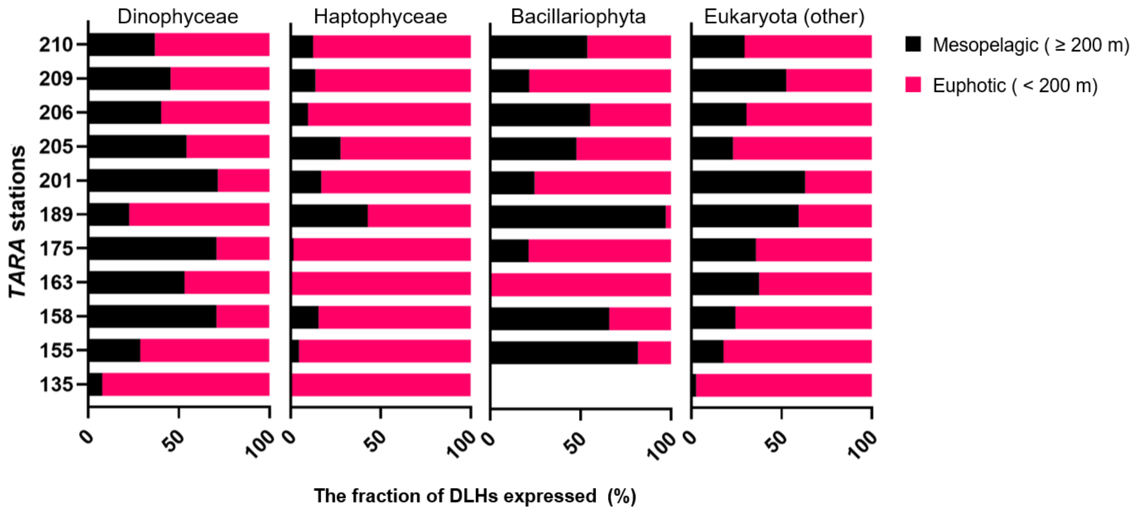

Fig. S8. Expression of DLHs in the euphotic versus mesopelagic zones in the *Tara* Ocean dataset. DLHs (DMSP lyase homologs) were detected in mesopelagic depths (200-751 m) in 11 stations (Y axis). The fraction of DLH transcripts expressed in the mesopelagic (black) and euphotic (pink) zone is presented for the main DLHs-expressing taxa.

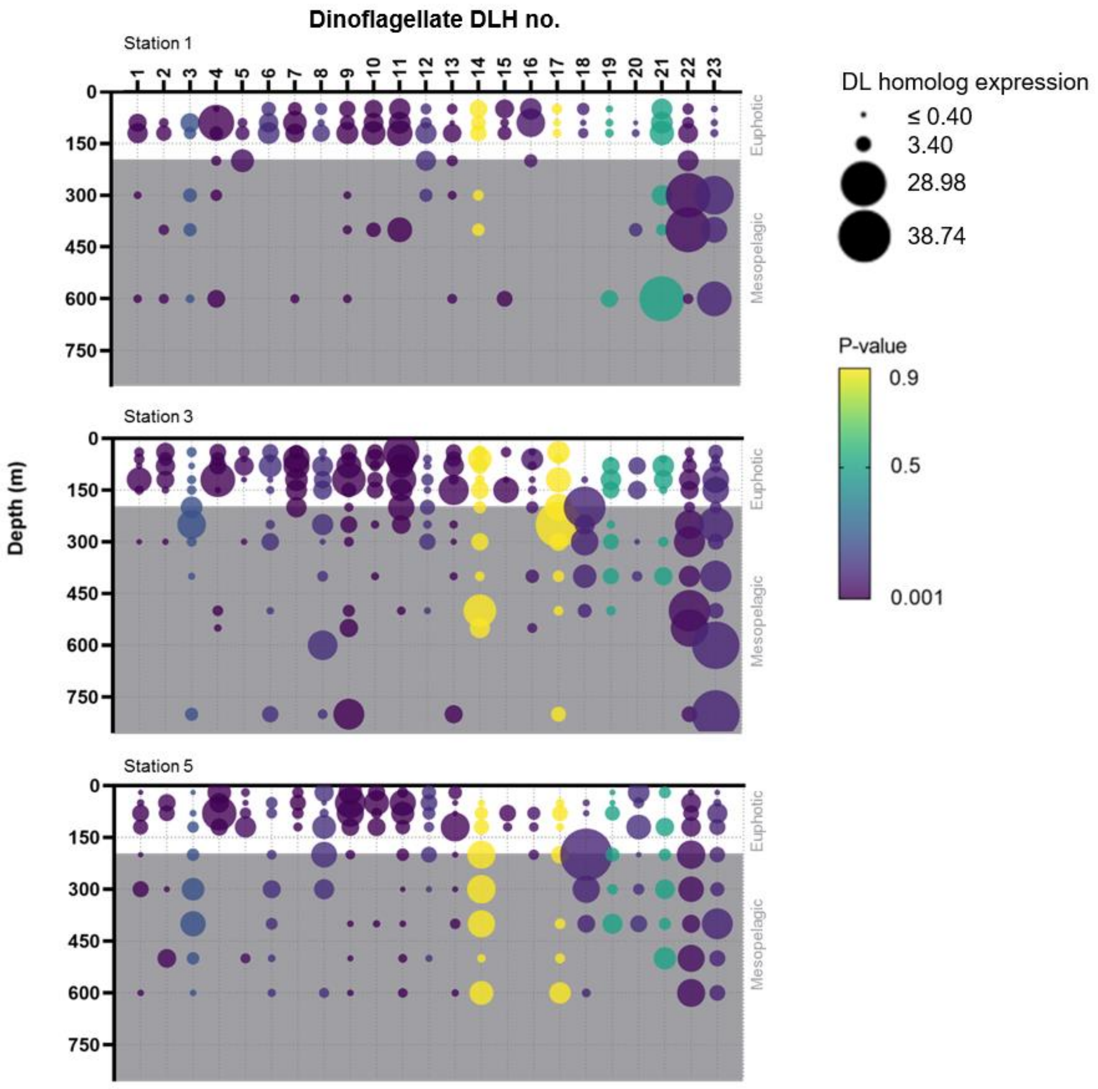

Fig. S9. Dinoflagellate DLHs are differentially expressed in euphotic and mesopelagic depths in the central Pacific Ocean. The expression level of 23 DLHs from natural dinoflagellates population is presented for three different locations (stations 1, 3, 5) in the central Pacific Ocean. Each station contains a depth profile. The circle size represents the normalized expression level. The color depicts the P - value, showing how significantly different is the expression between the euphotic (white background) and the mesopelagic (grey background) zones. The expression data was taken from Cohen NR, et al. (2021).

Table S1. Conserved motifs in the DL protein sequence.

Motif	P-value	E-value	Sites	Training set-positives	Training set-negatives	Score	Testing set-positives	Testing set-negatives	P-value	Match Threshold
GCZDVPGFD	1.40E-16	4.10E-15	145 (96.0%)	131 / 136 (96.3%)	0 / 826 (0.0%)	5.20E-157	14 / 15 (93.3%)	0 / 91 (0.0%)	1.40E-16	7.54392
GDCGFMMAFQ	6.60E-15	1.90E-13	149 (98.7%)	136 / 136 (100.0%)	0 / 826 (0.0%)	1.60E-169	13 / 15 (86.7%)	0 / 91 (0.0%)	6.60E-15	25.5612
IRAILLECTEL	6.60E-15	1.90E-13	148 (98.0%)	135 / 136 (99.3%)	0 / 826 (0.0%)	1.30E-166	13 / 15 (86.7%)	0 / 91 (0.0%)	6.60E-15	25.6013
EAKGVSGIT	6.60E-15	1.90E-13	126 (83.4%)	113 / 136 (83.1%)	3 / 826 (0.4%)	2.50E-119	13 / 15 (86.7%)	0 / 91 (0.0%)	6.60E-15	11.3761
RVVPGLTFEMAQSG	2.10E-13	6.00E-12	144 (95.4%)	132 / 136 (97.1%)	0 / 826 (0.0%)	3.20E-159	12 / 15 (80.0%)	0 / 91 (0.0%)	2.10E-13	16.3706
GVIRLDYBY	2.10E-13	6.00E-12	143 (94.7%)	131 / 136 (96.3%)	0 / 826 (0.0%)	5.20E-157	12 / 15 (80.0%)	0 / 91 (0.0%)	2.10E-13	15.5877
RAATGLPVFDAI	2.10E-13	6.00E-12	143 (94.7%)	131 / 136 (96.3%)	0 / 826 (0.0%)	5.20E-157	12 / 15 (80.0%)	0 / 91 (0.0%)	2.10E-13	20.2413
GDIDHPGSFGY	2.10E-13	6.00E-12	144 (95.4%)	132 / 136 (97.1%)	2 / 826 (0.2%)	2.80E-155	12 / 15 (80.0%)	0 / 91 (0.0%)	2.10E-13	21.5522
ILTANSKSLKP	6.70E-13	1.90E-11	147 (97.4%)	134 / 136 (98.5%)	0 / 826 (0.0%)	5.50E-164	13 / 15 (86.7%)	2 / 91 (2.2%)	6.70E-13	16.4622
PPYADAL	6.70E-13	1.90E-11	132 (87.4%)	119 / 136 (87.5%)	10 / 826 (1.2%)	4.20E-120	13 / 15 (86.7%)	2 / 91 (2.2%)	6.70E-13	13.6881

Table S2. DL homologs identified by Alcolombri et al., (2015). The WP accessions are from NCBI and the CAMPEP accessions are from the MMETSP databases.

Species	Domain	Phylum	Name in tree- Fig. 4a from Alcolombri et al., 2015	Accession number
Paraburkholderia ferrariae	Bacteria	Proteobacteria	P. ferrariae	WP_028225891.1
Streptosporangium roseum	Bacteria	Actinobacteria	S. roseum	WP_051865922.1
Robbsia andropogonis	Bacteria	Proteobacteria	R. andropongonis	WP_024904647.1
Geopsychrobacter electrodiphilus	Bacteria	Proteobacteria	G. electrodiphilus	WP_020674609.1
Azospirillum lipoferum	Bacteria	Proteobacteria	A. lipoferum	WP_042444357.1
Pseudovibrio sp. FO-BEG1	Bacteria	Proteobacteria	Pseudovibrio sp.	WP_014285483.1
Micromonospora sp. ATCC 39149	Bacteria	Actinomycetia	Micromonospora sp.	WP_007071402.1
Agrobacterium (Multispecies)	Bacteria	Proteobacteria	Agrobacterium multi	WP_007689264.1
Mesorhizobium loti	Bacteria	Proteobacteria	M. loti	WP_019863393.1
Actinomadura madurae	Bacteria	Actinobacteria	A. madurae	WP_063747721.1
Amphritea japonica	Bacteria	Proteobacteria	A. japonica	WP_019621467.1
Desulfotignum phosphitoxidans	Bacteria	Proteobacteria	D. phosphitoxidans	WP_006965703.1
Roseobacter sp. SK209-2-6	Bacteria	Proteobacteria	Roseobacter sp.	WP_008209391.1
unclassified Leisingera (Multispecies)	Bacteria	Proteobacteria	Leisingera multi	WP_019296982.1
Alkalihalobacillus wakoensis	Bacteria	Firmicutes	A. wakoensis	WP_034745099.1
Natranaerobius thermophilus	Bacteria	Firmicutes	N. thermophilus	WP_012448288.1
Desulfatirhabdium butyrativorans	Bacteria	Proteobacteria	D. butyrativorans	WP_028324949.1
Acetomicrobium hydrogeniformans	Bacteria	Synergistetes	A. hydrogeniformans	WP_009200906.1
Neptuniibacter caesariensis	Bacteria	Proteobacteria	N. caesariensis	WP_007022653.1
Agrobacterium rhizogenes	Bacteria	Proteobacteria	A. rhizogenes	WP_047469519.1
Candidatus Puniceispirillum marinum	Bacteria	Proteobacteria	C.P. marinum	WP_013045355.1
Crypthecodinium cohnii (Seligo)	Eukaryota	Dinoflagellata	C. cohnii A	CAMPEP_0193854358
Crypthecodinium cohnii (Seligo)	Eukaryota	Dinoflagellata	C. cohnii B	CAMPEP_0193926808
Crypthecodinium cohnii (Seligo)	Eukaryota	Dinoflagellata	C. cohnii C	CAMPEP_0193865556
Crypthecodinium cohnii (Seligo)	Eukaryota	Dinoflagellata	C. cohnii D	CAMPEP_0193933962
Azadinium spinosum 3D9	Eukaryota	Dinoflagellata	A. spinosum A	CAMPEP_0186837182
Azadinium spinosum 3D9	Eukaryota	Dinoflagellata	A. spinosum B	CAMPEP_0186768496
Karenia brevis CCMP2299	Eukaryota	Dinoflagellata	K. brevis A	CAMPEP_0188921804
Karenia brevis CCMP2299	Eukaryota	Dinoflagellata	K. brevis B	CAMPEP_0188855392
Karenia brevis CCMP2299	Eukaryota	Dinoflagellata	K. brevis C	CAMPEP_0188922230
Alexandrium temarense CCMP1771	Eukaryota	Dinoflagellata	A. temarense A	CAMPEP_0186397762
Alexandrium temarense CCMP1771	Eukaryota	Dinoflagellata	A. temarense B	CAMPEP_0186178466
Peridinium aciculiferum PAER_2	Eukaryota	Dinoflagellata	P. aciculiferum	CAMPEP_0190608078
Durinskia baltica CSIRO_CS-38	Eukaryota	Dinoflagellata	D. baltica	CAMPEP_0200062214
Amphidinium carterae CCMP1314	Eukaryota	Dinoflagellata	A. carterae A	CAMPEP_0186439658
Amphidinium carterae CCMP1314	Eukaryota	Dinoflagellata	A. carterae B	CAMPEP_0186460378
Glenodinium foliaceum CCAP1116	Eukaryota	Dinoflagellata	G. foliaceum A	CAMPEP_0188240084
Glenodinium foliaceum CCAP1116	Eukaryota	Dinoflagellata	G. foliaceum B	CAMPEP_0188434408
Isochrysis sp.	Eukaryota	Haptophyta	Isochrysis sp. A	CAMPEP_0188750792
Isochrysis sp.	Eukaryota	Haptophyta	Isochrysis sp. B	CAMPEP_0188739558
Prymnesium parvum Texoma	Eukaryota	Haptophyta	P. parvum A	CAMPEP_0191203590
Prymnesium parvum Texoma	Eukaryota	Haptophyta	P. parvum B	CAMPEP_0191252250

Table S3. Confirmation of DL homologs expression in the MMETSP database.

Species	MMETSP Sample IDs	Contigs	Expression ?
P. aciculiferum	MMETSP0370, MMETSP0371	Peridinium-aciculiferum-PAER_2-20130926|2150_0	yes
G. foliaceum	MMETSP0118_2, MMETSP0119_2	Glenodinium-foliaceum-CCAP1116_3-20130913|6335_1	yes
		Glenodinium-foliaceum-CCAP1116_3-20130913|269394_1	yes
		Glenodinium-foliaceum-CCAP1116_3-20130913|39119_1	yes
		Glenodinium-foliaceum-CCAP1116_3-20130913|264918_1	yes
		Glenodinium-foliaceum-CCAP1116_3-20130913|265187_1	yes
		Glenodinium-foliaceum-CCAP1116_3-20130913|162165_1	yes
		Glenodinium-foliaceum-CCAP1116_3-20130913|11173_1	yes
		Glenodinium-foliaceum-CCAP1116_3-20130913|144842_1	yes
S. kawagutii	MMETSP0132_2C, MMETSP0133_2, MMETSP0134_2, MMETSP0135_2	Symbiodinium-kawagutii-CCMP2468-20131203|3329_1	no
Symbiodinium-sp-C15	MMETSP1370, MMETSP1371	Symbiodinium-sp-C15-20130923|6074_1	yes
		Symbiodinium-sp-C15-20130923|119450_1	yes
		Symbiodinium-sp-C15-20130923|60114_1	yes
		Symbiodinium-sp-C15-20130923|18175_1	yes
		Symbiodinium-sp-C15-20130923|25623_1	yes
		Symbiodinium-sp-C15-20130923|18047_1	yes
Symbiodinium-sp-Mp	MMETSP1122, MMETSP1123, MMETSP1124, MMETSP1125	Symbiodinium-sp-Mp-20130822|28546_1	yes
		Symbiodinium-sp-Mp-20130822|90026_1	yes
		Symbiodinium-sp-Mp-20130822|7847_1	yes
		Symbiodinium-sp-Mp-20130822|75336_1	yes
		Symbiodinium-sp-Mp-20130822|189624_1	yes
Symbiodinium-sp-C1	MMETSP1367, MMETSP1369	Symbiodinium-sp-C1-20140214|19746_1	yes
		Symbiodinium-sp-C1-20140214|27762_1	yes
		Symbiodinium-sp-C1-20140214|794_1	yes
		Symbiodinium-sp-C1-20140214|25073_1	yes
		Symbiodinium-sp-C1-20140214|27039_1	yes
		Symbiodinium-sp-C1-20140214|22040_1	yes
K. brevis	MMETSP0027, MMETSP0029, MMETSP0030,	Karenia-brevis-CCMP2229-20130916|56488_1	yes
		Karenia-brevis-CCMP2229-20130916|7473_1	yes
		Karenia-brevis-CCMP2229-20130916|56810_1	yes
A. carterae	MMETSP0398C, MMETSP0399, MMETSP0258, MMETSP0259	Amphidinium-carterae-CCMP1314-20130924|22079_1	yes
		Amphidinium-carterae-CCMP1314-20130924|63059_1	yes, except MMETSP0399
		Amphidinium-carterae-CCMP1314-20130924|57390_1	only in MMETSP0258, MMETSP0259
		Amphidinium-carterae-CCMP1314-20130924|23864_1	yes
		Amphidinium-carterae-CCMP1314-20130924|16687_1	only in MMETSP0258, MMETSP0259
C. cohnii	MMETSP0323_2, MMETSP0324_2, MMETSP0325_2, MMETSP0326_2	Crypthecodinium-cohnii-Seligo-20130904|11054_1	yes
		Crypthecodinium-cohnii-Seligo-20130904|174461_1	yes
		Crypthecodinium-cohnii-Seligo-20130904|19770_1	yes
		Crypthecodinium-cohnii-Seligo-20130904|195906_1	yes, except MMETSP0323_2
		Crypthecodinium-cohnii-Seligo-20130904|20414_1	yes
A. spinosum	MMETSP1036_2, MMETSP1037_2, MMETSP1038_2	Azadinium-spinosum-3D9-20130829|183522_1	yes
		Azadinium-spinosum-3D9-20130829|29318_1	yes
		Azadinium-spinosum-3D9-20130829|17069_1	yes
		Azadinium-spinosum-3D9-20130829|21942_1	yes
D. baltica	MMETSP0116_2, MMETSP0117_2	Durinskia-baltica-CSIRO_CS-38-20140214|203657_1	no
		Durinskia-baltica-CSIRO_CS-38-20140214|157896_1	yes
		Durinskia-baltica-CSIRO_CS-38-20140214|5581_1	yes
		Durinskia-baltica-CSIRO_CS-38-20140214|164224_1	yes
		Durinskia-baltica-CSIRO_CS-38-20140214|160103_1	yes
		Durinskia-baltica-CSIRO_CS-38-20140214|157677_1	yes
		Durinskia-baltica-CSIRO_CS-38-20140214|238428_1	yes
		Durinskia-baltica-CSIRO_CS-38-20140214|230672_1	yes
		Durinskia-baltica-CSIRO_CS-38-20140214|6229_1	yes
		Durinskia-baltica-CSIRO_CS-38-20140214|209311_1	only in MMETSP0117_2
		Durinskia-baltica-CSIRO_CS-38-20140214|151706_1	yes
		Durinskia-baltica-CSIRO_CS-38-20140214|238089_1	yes
		Durinskia-baltica-CSIRO_CS-38-20140214|14482_1	yes
A. temarense	MMETSP0378, MMETSP0380, MMETSP0382, MMETSP0384	Alexandrium-temarense-CCMP1771-20130823|410328_1	yes
		Alexandrium-temarense-CCMP1771-20130823|2738_1	yes
		Alexandrium-temarense-CCMP1771-20130823|37484_1	yes
		Alexandrium-temarense-CCMP1771-20130823|165856_1	yes

Table S3- continue. Confirmation of DL homologs expression in the MMETSP database.

Isochrysis sp.	MMETSP1388, MMETSP1090	Isochrysis-sp-CCMP1244-20130912|8451_1	yes
		Isochrysis-sp-CCMP1244-20130912|1392_1	yes
		Isochrysis-sp-CCMP1244-20130912|2246_1	yes
		Isochrysis-sp-CCMP1244-20130912|23305_1	yes
E. huxleyi CCMP370	MMETSP1154, MMETSP1155, MMETSP1156, MMETSP1157	Emiliania-huxleyi-CCMP370-20130905|29607_1	yes
E. huxleyi PLYM219	MMETSP1150, MMETSP1151, MMETSP1152, MMETSP1153	Emiliania-huxleyi-PLYM219-20130905|8612_1	yes
		Emiliania-huxleyi-PLYM219-20130905|91030_1	yes
E. huxleyi CCMP379	MMETSP0994, MMETSP0995, MMETSP0996, MMETSP0997	Emiliania-huxleyi-379-20130905|8113_1	yes
		Emiliania-huxleyi-379-20130905|18426_1	yes
C. polylepis	MMETSP0143, MMETSP0145,	Chrysochromulina-polylepis-CCMP1757-20130903|18714_1	yes
P. parvum texoma	MMETSP0006_2, MMETSP0007, MMETSP0008_2, MMETSP0814,	Prymnesium-parvum-Texoma1-20131001|4355_1	yes
		Prymnesium-parvum-Texoma1-20131001|104153_1	yes
		Prymnesium-parvum-Texoma1-20131001|200449_1	yes
		Prymnesium-parvum-Texoma1-20131001|21691_1	yes
S. marinoi	MMETSP0918, MMETSP0920	Skeletonema-marinoi-SkelA-20130924|7776_1	yes
S. dohrnii	MMETSP0562, MMETSP0563	Skeletonema-dohrnii-SkelB-20130926|18976_1	yes
*P. fradulenta**	MMETSP0850, MMETSP0851,	Pseudo_nitzschia-fradulenta-WWA7-20140214|209615_1	only in MMETSP0851
		Pseudo_nitzschia-fradulenta-WWA7-20140214|96008_1	only in MMETSP0851
*T.antarctica**	MMETSP0902, MMETSP0903,	Thalassiosira-antarctica-CCMP982-20140214|20162_1	yes
*Tx. antarctica**	MMETSP0152, MMETSP0154	Thalassiothrix-antarctica-L6_D1-20140214|17297_1	yes
*F. kerguelensis**	MMETSP0906, MMETSP0907, MMETSP0908 MMETSP0909	Fragilariopsis-kerguelensis-L2_C3-20140214|35188_1	yes
T. striata	MMETSP0817, MMETSP0818, MMETSP0819, MMETSP0820	Tetraselmis-striata-LANL1001-20140214|2880_1	yes
		Tetraselmis-striata-LANL1001-20140214|11490_1	yes
		Tetraselmis-striata-LANL1001-20140214|18675_1	yes, except in MMETSP0817
		Tetraselmis-striata-LANL1001-20140214|95585_1	yes, except in MMETSP0817

*Note: No genome was available for this species, the putative homolog was found in the MMETSP transcriptome data only.

Table S4. Expression levels of identified DLHs under environmental stress conditions.

Species	DL homolog	Experiment (stress condition tested)	DL expression - control	DL expression - stress condition tested	DL expression- Fold change	Biological replicates	Source
Azadinium spinosum	Azadinium spinosum A	Cold stress	0	2710	-	1	MMETSP
	Azadinium spinosum B	Cold stress	203	191	0.9	1	MMETSP
	Azadinium spinosum C	Cold stress	685	702	1.0	1	MMETSP
	Azadinium spinosum D	Cold stress	1048	1115	1.1	1	MMETSP
Amphidinium carterae	Amphidinium carterae A	High light	12	16	1.3	1	MMETSP
	Amphidinium carterae B	High light	0	0	not expressed	1	MMETSP
	Amphidinium carterae C	High light	0	7	-	1	MMETSP
	Amphidinium carterae D	High light	196	199	1.0	1	MMETSP
	Amphidinium carterae E	High light	0	0	not expressed	1	MMETSP
Isochrysis	Isochrysis sp. A	High light	334	316	0.95	1	MMETSP
	Isochrysis sp. B	High light	194	490	2.53	1	MMETSP
	Isochrysis sp. C	High light	89	33865	382.46	1	MMETSP
	Isochrysis sp. D	High light	1597	846	0.53	1	MMETSP
Thalassiosira antarctica	T.antarctica A	Cold stress	203	399	1.97	control -2; cold stress-1	MMETSP
	T. antarctica B	Cold stress	30	18	0.59	control -2; cold stress-1	MMETSP
Thalassiosira antarctica	T.antarctica A	Si limitation	440	19	0.04	control -2; Si limitation-1	MMETSP
	T. antarctica B	Si limitation	31	0	0.00	control -2; Si limitation-1	MMETSP
Pseudonitzschia fradulenta	P, fradulenta A	Si limitation	0	260	-	1	MMETSP
	P. fradulenta B	Si limitation	0	568	-	1	MMETSP
Skeletonema dohrnii	S. dohrnii	N limitation	73	11	0.15	1	MMETSP
Seminavis robusta	S. robusta	N limitation (48 h)	4	2	0.54	2	Osuna-Cruz et al., (2020)
	S. robusta	N limitation (72 h)	10	1	0.10	2	Osuna-Cruz et al., (2020)
Ulva mutabilis	U. mutabilis A	Cold stress	Not available	Not available	no change	4	De Clerck et al., (2018)
	U. mutabilis B	Cold stress	Not available	Not available	0.08	8	De Clerck et al., (2018)
Acropora millepora	A. millepora A	High CO2	3453	2919	0.85	3	Moya et al., (2012)
	A. millepora D	High CO2	5850	5102	0.87	3	Moya et al., (2012)
	A. millepora E	High CO2	1999	1497	0.75	3	Moya et al., (2012)
	A. millepora F	High CO2	6066	6202	1.02	3	Moya et al., (2012)
	A. millepora I	High CO2	10976	10177	0.93	3	Moya et al., (2012)
	A. millepora G	High CO2	4221	3400	0.81	3	Moya et al., (2012)

Table S5. DL homolog expression level and DMS concentration in surface water.

DLH expression data					DMS data from Hulswar et al., 2022			
***Tara* station**	**Date of sampling**	**Latitude**	**Longitude**	**Total DLH transcripts in all size fractions (relative abundanc)**	**Month**	**Latitude**	**Longitude**	**Monthely average of DMS con. (nM)**
TARA_004	9/15/2009	36.553	-6.567	1.12E-04	September	37	-7	2.452
TARA_007	9/23/2009	37.040	1.948	8.94E-05	September	37	2	2.403
TARA_009	9/28/2009	39.124	5.854	3.15E-05	September	39	6	2.482
TARA_018	11/2/2009	35.754	14.274	8.78E-05	November	36	14	2.314
TARA_020	11/12/2009	34.430	14.998	1.25E-05	November	34	15	2.314
TARA_022	11/16/2009	39.831	17.410	1.05E-04	November	40	17	2.314
TARA_023	11/18/2009	42.190	17.717	1.25E-04	November	42	18	2.314
TARA_025	11/23/2009	39.345	19.394	8.12E-05	November	39	19	2.314
TARA_026	11/24/2009	38.475	20.171	4.91E-05	November	38	20	2.314
TARA_030	12/15/2009	33.918	32.881	2.60E-05	December	34	33	2.251
TARA_036	3/12/2010	20.819	63.507	6.72E-05	March	21	64	3.168
TARA_038	3/15/2010	19.038	64.498	4.94E-05	March	19	64	3.218
TARA_039	3/18/2010	18.581	66.567	8.62E-05	March	19	67	3.111
TARA_040	3/22/2010	17.529	67.958	1.05E-04	March	18	68	2.954
TARA_041	3/30/2010	14.574	69.999	5.22E-05	March	15	70	2.816
TARA_046	4/15/2010	-0.662	73.163	6.59E-05	April	-1	73	2.392
TARA_047	4/16/2010	-2.044	72.161	3.41E-05	April	-2	72	2.373
TARA_048	4/19/2010	-9.415	66.303	2.28E-05	April	-9	66	1.953
TARA_051	5/11/2010	-21.485	54.333	1.25E-04	May	-21	54	2.817
TARA_052	5/17/2010	-16.961	53.962	1.36E-04	May	-17	54	2.768
TARA_064	7/7/2010	-29.500	37.991	1.22E-04	July	-30	38	3.838
TARA_065	7/12/2010	-35.190	26.291	7.03E-05	July	-35	26	3.050
TARA_066	7/15/2010	-34.931	17.951	6.02E-05	July	-35	18	3.115
TARA_067	9/7/2010	-32.199	17.705	7.37E-05	September	-32	18	3.263
TARA_068	9/14/2010	-31.047	4.667	9.54E-05	September	-31	5	1.716
TARA_070	9/21/2010	-20.405	-3.185	1.27E-04	September	-20	-3	1.338
TARA_072	10/5/2010	-8.750	-17.919	5.83E-05	October	-9	-18	1.715
TARA_076	10/16/2010	-20.950	-35.219	1.24E-04	October	-21	-35	0.862
TARA_078	11/4/2010	-30.173	-43.284	1.36E-04	November	-30	-43	1.959
TARA_080	11/29/2010	-40.645	-52.141	1.43E-04	November	-41	-52	4.830
TARA_081	12/2/2010	-44.579	-52.303	7.58E-05	December	-45	-52	3.174
TARA_082	12/6/2010	-47.192	-58.258	2.71E-04	December	-47	-58	3.258
TARA_083	12/16/2010	-54.376	-65.113	1.10E-04	December	-54	-65	2.994
TARA_084	1/3/2011	-60.284	-60.588	1.25E-04	January	-60	-61	1.653
TARA_085	1/6/2011	-62.074	-49.460	4.53E-04	January	-62	-49	2.357
TARA_092	2/26/2011	-33.684	-71.984	1.86E-04	February	-34	-72	1.222
TARA_093	3/12/2011	-34.057	-73.103	5.21E-05	March	-34	-73	2.305
TARA_098	4/2/2011	-25.808	-111.730	1.01E-04	April	-26	-112	1.227

TARA_100	4/14/2011	-12.976	-96.002	2.06E-04	April	-13	-96	2.014
TARA_102	4/21/2011	-5.253	-85.181	1.27E-04	April	-5	-85	3.250
TARA_109	5/12/2011	2.016	-84.569	1.25E-04	May	2	-85	3.303
TARA_110	4/14/2011	-1.991	-84.595	1.08E-04	April	-2	-85	4.132
TARA_111	5/31/2011	-16.958	-100.654	1.53E-04	May	-17	-101	1.612
TARA_122	7/26/2011	-8.998	-139.219	1.54E-04	July	-9	-139	2.284
TARA_123	7/31/2011	-8.902	-140.288	1.67E-04	July	-9	-140	2.284
TARA_124	8/4/2011	-9.150	-140.516	1.32E-04	August	-9	-141	2.205
TARA_125	8/8/2011	-8.904	-142.588	1.38E-04	August	-9	-143	2.205
TARA_128	9/4/2011	0.007	-153.686	1.32E-04	September	0	-154	2.459
TARA_131	9/29/2011	22.741	-157.979	1.84E-04	September	23	-158	1.563
TARA_132	10/4/2011	31.504	-159.035	1.97E-04	October	32	-159	1.398
TARA_135	10/23/2011	33.005	-121.782	1.26E-04	October	33	-122	1.717
TARA_136	11/30/2011	17.064	-118.899	1.26E-04	November	17	-119	1.287
TARA_137	12/2/2011	14.197	-116.645	1.07E-04	December	14	-117	2.455
TARA_138	12/10/2011	6.327	-102.953	6.26E-05	December	6	-103	3.346
TARA_139	12/15/2011	6.494	-94.981	9.28E-05	December	6	-95	3.265
TARA_142	1/9/2012	25.550	-88.407	1.09E-04	January	26	-88	2.698
TARA_143	1/16/2012	29.724	-79.629	3.96E-05	January	30	-80	1.400
TARA_144	1/29/2012	36.354	-72.844	6.74E-05	January	36	-73	0.926
TARA_145	2/2/2012	39.217	-70.038	1.69E-04	February	39	-70	1.370
TARA_146	2/15/2012	34.763	-71.246	1.25E-04	February	35	-71	1.572
TARA_147	2/18/2012	33.020	-66.551	1.88E-04	February	33	-67	1.325
TARA_148	2/24/2012	31.760	-64.183	1.39E-04	February	32	-64	1.300
TARA_149	3/1/2012	34.067	-49.850	4.49E-05	March	34	-50	1.117
TARA_150	3/5/2012	35.908	-37.261	1.37E-04	March	36	-37	1.114
TARA_151	3/9/2012	36.159	-29.006	1.58E-04	March	36	-29	1.277
TARA_152	3/19/2012	43.683	-16.842	1.39E-04	March	44	-17	1.417
TARA_155	5/24/2013	54.547	-16.889	1.47E-04	May	55	-17	5.955
TARA_158	6/3/2013	67.130	0.298	4.99E-05	June	67	0	7.320
TARA_163	6/9/2013	76.184	1.457	2.32E-03	June	76	1	6.805
TARA_168	7/1/2013	72.566	44.111	1.26E-04	July	73	44	4.521
TARA_173	7/8/2013	78.945	79.361	1.90E-04	July	79	79	1.312
TARA_175	7/10/2013	78.568	68.688	8.13E-04	July	79	69	1.354
TARA_178	7/18/2013	75.981	74.677	7.39E-05	July	76	75	1.688
TARA_180	7/18/2013	76.528	83.995	1.34E-04	July	77	84	1.906
TARA_188	8/15/2013	78.305	91.719	2.50E-03	August	78	92	2.010
TARA_189	8/27/2013	77.920	117.017	1.10E-04	August	78	117	1.946
TARA_191	9/3/2013	71.556	160.941	1.90E-05	September	72	161	0.844
TARA_193	9/8/2013	71.099	174.928	6.80E-05	September	71	175	0.985
TARA_194	9/11/2013	73.382	-168.205	5.05E-05	September	73	-168	1.535
TARA_196	9/14/2013	71.907	-154.920	6.12E-05	September	72	-155	1.974
TARA_201	9/30/2013	74.312	-85.849	6.42E-05	September	74	-86	0.844
TARA_205	10/8/2013	72.461	-71.892	8.99E-05	October	72	-72	0.485
TARA_206	10/12/2013	70.956	-53.576	9.43E-05	October	71	-54	0.437
TARA_208	10/20/2013	69.116	-51.550	8.23E-05	October	69	-52	0.437
TARA_209	10/23/2013	64.736	-53.060	1.39E-04	October	65	-53	0.359
TARA_210	10/27/2013	61.533	-55.992	1.40E-04	October	62	-56	0.280

Dataset S1 (separate file). DMSP lyase homologs.

Dataset S2 (separate file). Amino acid sequences of predicted DL homologs.

Dataset S3 (separate file). Species with no DL homologs identified in available databases.
